# Utility of renal resistive index as an early marker of acute kidney injury related to intra-abdominal pressure in critical ill patients

**DOI:** 10.1186/2197-425X-3-S1-A461

**Published:** 2015-10-01

**Authors:** P Ortiz Ballujera, C Vera Ching, J Calabia Martinez, C Molina Rodriguez, A Marin Valencia, J Gonzalez Londoño, JM Sirvent

**Affiliations:** Hospital Universitario Dr. Josep Trueta, Intensive Care Unit, Girona, Spain; Hospital Universitario Dr. Josep Trueta, Nephrology, Girona Spain

## Introduction

There is a direct relationship between intra-abdominal hypertension (IAH) and acute kidney injury (AKI). The current AKI scales are not always an early marker of renal damage. The use of renal resistive index (RI) measured by eco-Doppler method for early detection of AKI in critically ill patients is postulated. The RI is a parameter obtained from the study of the flow of the arcuate or renal interlobar arteries. The formula is: RI = (systolic peak velocity -diastolic end velocity) / systolic peak velocity. The definite result is an average of 3-5 pulse waves.

## Objectives

Our aim is to assess the usefulness of RI by eco- Doppler as an early marker of acute renal failure in patients with risk of IAH.

## Methods

We conducted a prospective observational study between April 2013 and June 2014. The inclusion criteria for our study were patients (age>18 years) admitted in intensive care unit ( ICU) with at least 3 IAH risk factors. Clinical and laboratory variables from all individuals were collected and the IAP (Intra-Abdominal Pressure) was monitored intermittently with a bladder placement catheter (UnoMeter Abdo-Pressure™). Further on, renal Doppler sonography was performed after inclusion and at least one more posterior determination was done, in order to measure the RI. The exclusion criteria were chronic renal failure history and / or risk factors for altered RI such as dilated urinary tract, renal artery pathology and renal transplant.

## Results

A total of 25 patients were included: 20 patients were men; average age 52 years (interquartile range 43.5-64), the median IAP was 12 mmHg (10.9-14), the median creatinine level was 0.99 mg/dL (0.59- 2.07). The reasons of ICU admission were: Sepsis 28%, postoperative abdominal surgery 28%, pancreatitis 16%, polytrauma 12%, peritonitis 12% and hemorrhagic shock 4%. Patients who developed AKI showed higher initial RI (0.68 vs 0.61, p = 0.016). We found positive correlation between RI and creatinine levels (p = 0.004).

From the 14 patients who presented IAH, AKI was present in 10 cases. From the 11 patients without IAH, AKI was present in 7 cases (no significant differences were found).

No correlation could be established between IAH and the RI value or AKI.

## Conclusions

According to our study, renal resistive index (RI) increase is a predictor of Acute Kidney Injury (AKI).

No correlation was found between Intra-Abdominal Hypertension (IAH) and the RI value or between IAH and AKI.

